# Retraction: MiR-486-3p inhibits the proliferation, migration and invasion of retinoblastoma cells by targeting ECM1

**DOI:** 10.1042/BSR-2020-0392_RET

**Published:** 2023-02-10

**Keywords:** ECM1, Migration, miR-486-3p, Proliferation, Retinoblastoma

This article is being retracted from *Bioscience Reports* at the request of the Editor-in-Chief and the Editorial Board following receipt of a notification from a reader, alerting the Editorial Board to concerns regarding the western blots and flow-cytometry plots of this manuscript. The western blots have concerning features such as the shapes of the bands and absence of any detectable background, and the flow-cytometry plots of Figure 6E share strong similarities with those found in other papers by unrelated authors, including in Figures 2E and 4F of Li et al. 2019 (https://doi.org/10.1080/15384101.2019.1674071). The authors have been contacted with regards to these concerns and subsequently the retraction, but they have not responded to the Journal's queries or the concerns raised. Given the extent of the issues raised, the Editorial Board stand by the decision to retract the article.

